# Gender and Racial Differences in the Cardiovascular Risk Factors among Overweight and Obese Rural Adults, Kuching and Samarahan Division, Sarawak, Malaysia

**DOI:** 10.1155/2016/4536753

**Published:** 2016-11-09

**Authors:** Whye Lian Cheah, Ching Thon Chang, Helmy Hazmi, Wan Manan Wan Muda

**Affiliations:** ^1^Department of Community Medicine & Public Health, Faculty of Medicine & Health Sciences, Universiti Malaysia Sarawak, Kota Samarahan, Sarawak, Malaysia; ^2^Department of Nursing, Faculty of Medicine & Health Sciences, Universiti Malaysia Sarawak, Kota Samarahan, Sarawak, Malaysia; ^3^School of Health Sciences, Universiti Sains Malaysia, Kubang Kerian, Kelantan, Malaysia

## Abstract

*Objective*. This study aimed to determine whether gender and ethnic differences had an effect on cardiovascular risk factors in overweight and obese rural adults in Sarawak.* Design and Setting*. This was a cross-sectional study conducted in rural communities in Kuching and Samarahan division, Malaysia. Data was obtained using a set of questionnaire (sociodemographic data and physical activity), measurement of blood pressure, height, weight (body mass index, BMI), body fat percentage, fasting blood sugar, and lipid profile from three ethnic groups—Iban, Malay, and Bidayuh. Analysis of data was done using SPSS version 23.0.* Results*. A total of 155 respondents participated in the study (81.6% response rate). The levels of physical activity, BMI status, body fat, hypercholesterolemia, and hyperglycemia were similar across the three ethnic groups and both females and males. Iban and Bidayuh had significant higher Atherogenic Index of Plasma (AIP) when compared to the Malay (Bidayuh OR = 0.30, 95% CI 0.12, 0.78; Iban OR = 0.29, 95% CI 0.12, 0.69).* Conclusions.* The relationship between cardiovascular risk factors varied according to ethnic groups and gender. A better understanding of these differences would help in the design and implementation of intervention programme for the prevention of cardiovascular disease.

## 1. Introduction

World Health Organization estimated that 17.5 million of people die from cardiovascular diseases (CVD) each year constituting 31% of all deaths worldwide. Among the CVD deaths, more than three quarters are from low-income and middle-income countries [1]. The risk factors of CVD have not changed much over the years. The evolution of treatment modalities and the intense public health preventive efforts has reduced the influence of smoking, untreated hypertension, and hypercholesterolaemia. However, sedentary lifestyle and overconsumption of processed and energy dense food products of poor nutritional values are on the increasing trend [2].

Although past studies have reported the role of obesity in the occurrence of CVD, recent studies have focused on body fat itself which give more distinct impact on CVD [2]. However, the relationship between body fat and the CVD risk factors is complicated by the dynamic influence and the mediating roles of the lipid parameters—triglycerides, HDL, and LDL [3].

Malaysia, with its rapid urbanization and changing lifestyle, is facing an increase in noncommunicable diseases (NCD) which resulted in the rise of mortality due to CVD from 15.7% in 1996 to 25.4% in 2006 [4, 5]. A study in a rural community in Sarawak among three major ethnic groups (Malay, Bidayuh, and Iban) reported the prevalence of overweight and obesity to be 39.6% and 11.9%, respectively. It further reported that, overall, 13% had hypertension and 1.5% had unhealthy random blood sugar reading above the national guideline level of 11.1 mmol/L [6].

Previous studies have identified the effect of gender, race/ethnicity on lipid profile, and body fat where both gender and ethnicity have been linked to the differences in body composition and lipid profiles among the females [7]. Another study has shown that there were significant differences in the muscle mass, fat distribution, bone mass, and modulation of lipid profiles between non-Hispanic black and white women [8] and Hispanic and black women tend to accumulate body fat more than their white counterpart [9]. The differences in fat composition and lipid profile among major ethnic groups in Malaysia were reported by Rampal et al. [10] where indigenous groups in Sarawak have the highest prevalence in raised triglycerides and blood pressure and the lowest in HDL cholesterol. However the findings only reported indigenous groups in Sarawak as a whole, in which there are more than 40 subethnic groups with different languages, culture, and lifestyle. The major ethnic groups residing in rural areas of Sarawak are the Iban, the Bidayuh, and the Malay. This study aimed to determine whether gender and ethnic differences have an effect on cardiovascular risk factors among these three ethnic overweight and obese adults in Sarawak.

## 2. Methods

This was a cross-sectional study which included rural communities in Kuching and Samarahan division. Only these two divisions were chosen as majority of the Bidayuhs reside in these two divisions [11]. Kuching division consists of three districts (Kuching, Lundu, and Bau), whereas Samarahan division consists of four districts (Samarahan, Asajaya, Serian, and Simunjan). Based on the list obtained from the state district office, three villages with the largest population from each division were randomly selected. With the help of the respective Sarawak Administration Officers, informed consent forms were sent out to the head of each village to invite all the villagers. The inclusion criteria for respondents were age between 18 to 65 years; either overweight (body mass index [BMI] 25 to 29.9 kg/m^2^) or obese (BMI ≥30 kg/m^2^), physically capable, and not intellectually challenged. Sample size was calculated based on prevalence of obesity 14.3 [6], with 95% confidence rate and 10% nonresponse; estimated minimum sample size needed was 204. After screening all the villagers, 190 respondents fulfilled the inclusion criteria. However, only 155 respondents consented to join the study.

The data were collected using a set of questionnaire that asked for sociodemographic data, level of physical activity, measurement of blood pressure (BP), body mass index (BMI), body fat percentage, fasting blood sugar, and lipid profile. Blood pressure was measured using an Accoson mercury sphygmomanometer (AC Cossor & Son [Surgical] Ltd., Essex, UK). Two sets of reading were taken to obtain the average blood pressure. Level of physical activity was assessed using a validated translated Malay version of International Physical Activity Questionnaire (IPAQ) long form [12]. The classification of physical activity level is based on computation of MET-minutes according to IPAQ procedure.

Height was measured using a stadiometer (SECA, UK) model 213. Respondents were asked to stand on the body meter without footwear, and the measuring beam was adjusted to rest on top the respondent's head. The reading was taken nearest to 0.1 cm. Tanita Body Composition Analyser (SC-204) was used to obtain weight and body fat percentage. Body fat consists of both essential fat which is stored in small amounts to protect the body and adipose tissue that provides cushion and insulation for the whole body [2]. In this study, body fat percentage refers to the amount of body fat mass in regard to the total body weight. The analyser also generates information on BMI. Blood samples were collected by trained laboratory assisted and analysed in a certified private laboratory hired under the grant. Calculation of Atherogenic Index of Plasma (AIP) was based on 10 log of the ratio of the concentration of triglyceride (TG) to high-density lipoprotein cholesterol (HDL-C), where each concentration is expressed in mmol/L [13]. AIP values of 0.1 and below are associated with low and above 0.1 with medium and high cardiovascular risk [14]. Classification of fasting total cholesterol and glucose is based on NCEP ATPIII [15] and the Malaysian Diabetes Mellitus Guideline 2009 [16]. Based on these guidelines, fasting total cholesterol of more than 5.2 mmol/L is classified as borderline high. As for blood glucose, any reading of more than 5.6 mmol/L is classified as hyperglycemia.

Data was analysed using SPSS 23.0. Descriptive analyses such as mean and standard deviations, frequency, and percentages for all variables were presented. Binary logistic regression analyses were performed to identify gender and race associated with cardiovascular risk factors. A confidence interval of 95% and the *p* value of less than 0.05 were regarded as statistically significant.

Ethical approval was granted from the Human Research Ethics Committee of Universiti Sains Malaysia (ref.: USMKK/PPP/JEPeM[246.3(6)]) and conformed to the requirements for ethical procedures for research in Malaysia. Each respondent was briefed on the study and signed the informed consent.

## 3. Results

A total of 155 respondents participated in the study with the highest proportion being the Bidayuh (response rate of 81.6%). A preliminary univariate analysis was performed to determine the effect size of the study where Cohen's *d* was reported to be 0.7 and effect size of 0.33, indicating a medium to large effect size. Majority of them were Christian (71.0%). The details on the sociodemographic information are in Table 1.

Table 2 showed the health profile of the respondents and the differences between gender and three ethnic groups. Physical activity level, BMI status, body fat, hypercholesterolemia, and hyperglycemia did not differ across the three ethnic groups, as well as between male and female respondents. However, the Iban had significant higher mean AIP compared to Malay and Bidayuh (*p* = 0.004). The Malays were found to have the highest percentage of hypertension compared to other races (*p* = 0.02). Between male and female respondents, male respondents have higher percentage of intermediate and high AIP (*p* = 0.019).

Logistic regression was used to determine the prevalence of CVD risk factors by race, age, and gender (refer to Table 3). All the full models containing the CVD risk factors (AIP, body fat, physical activity, hypertension, hypercholesterolemia, and hyperglycemia) were statistically significant with *p* < 0.001, indicating that the models were able to distinguish between respondents with CVD risk factors and those without. The findings showed only race had significant association with AIP. The odds ratio or exp⁡(*B*) value for Bidayuh was 0.3, indicating Bidayuh respondents were 0.3 times more likely to have intermediate and high AIP than Malay respondents. Similarly for Iban respondents the odds ratio or exp⁡(*B*) value was 0.29, indicating Iban respondents were 0.29 times more likely to have intermediate and high AIP than Malay respondents. The Wald values of the independents variables indicated that race was significant predictor of AIP.

## 4. Discussion

Consistent with BMI, body fat percentage among respondents in this study was found to be at very unhealthy level with the mean percentage above 36% for all ethnic groups. Similarly for gender more than 94% of both males and females were found to be overfat. The mean fasting cholesterol, LDL, and triglyceride were also above the normal limits, indicating higher disease risk. Excess body fat has been shown to be related to diabetes, cardiovascular disease, metabolic syndrome, and other noncommunicable chronic diseases [17]. Such finding is alarming, particularly in rural communities where most of the respondents are involved in farming activities.

There is a significant age-adjusted racial difference in the prevalence of intermediate and high AIP among the respondents. Bidayuh and Iban respondents had higher prevalence of AIP compared to Malay respondents. Based on the descriptive results (Table 2), Malay respondents may have the highest mean BMI, body fat percentage, and lipid profile (except triglyceride) compared to Iban and Bidayuh respondents, but their AIP level was the lowest, indicating higher AIP might not be associated with higher BMI, body fat percentage, and other lipid indicators. These findings are not consistent with previous studies [18].

It is important to note that although individual lipid profile reflects cardiovascular risk, it is not as accurate as AIP level which has a stronger sensitivity that reflects the interaction between atherogenic and protective lipoprotein [19]. The mathematical formula involved the logarithmic transformation of triglyceride/HDL which is closely associated with LDL which is found to be a strong indicator in atherogenic lipoprotein phenotype [20]. This could explain why Iban have seemingly lower cholesterol and LDL and were at highest risk of cardiovascular disease based on AIP. Another possible explanation is that this community might not consume the right type of fat in their diet. This phenomenon is common in rural community where consumption of food is based on availability and affordability. A focus group study carried out among the overweight and obese Iban community reported that among the underlying cause of overweight and obesity was lack of knowledge on proper eating [21]. A study carried out among the reproductive-aged women showed significant ethnic differences between body fat distribution and serum lipid profiles. It was further argued that such differences could be due to difference in adipose tissue metabolism or lipid-clearance capacity and production [22]. Genetics and environmental factors may have also played a role in determining the differences in AIP [22].

Although cardiovascular risk factors such as lipid profile and body fat remain the major cause of death for both women and men in the world, literatures had showed that there are significant differences in gender differences in the prevalence of cardiovascular risk factors [23]. It is important to note that statistics had also showed that more men are living with and dying of cardiovascular related diseases than women but such pattern is only observed when at the age of 75 years [23]. Being the gender that has longer life expectancy, women would have lower mortality and morbidity rate than men [24]. It is assumed that the endogenous oestrogen during the fertile period of female life delays the manifestation of atherosclerosis [24]. However, what is alarming now is that the rate of cardiovascular related diseases among younger women aged 35 to 44 years has increased significantly on an average of 1.3% annually over a period of five years between 1997 and 2002 [25]. The findings of this study indicated otherwise that no significant difference was found between male and female respondents in all cardiovascular risk factors.

## 5. Conclusion

The limitation of this study was the use of a cross-sectional study design, which could not explain the causal relationship between variables. The small sample size may have limited the generalization of the results. Nevertheless, the findings of this study indicated the need to address obesity pandemic more seriously, particularly in the rural communities where detection of disease is still lacking. AIP can serve as a reliable indicator for higher risk of cardiovascular disease when the individual lipoproteins seem normal but with elevated triglyceride concentration. The relationship between lipid profiles and body fat varies according to gender and different ethnic groups. A better understanding of these differences in their health profile would help in the design and implementation of intervention programme in the prevention of cardiovascular disease. For future research, a bigger sample size that covers more divisions in Sarawak together with the adoption of more analytical study design such as prospective cohort or case-control study is recommended. This will help to generate a clearer picture on the potential risk factors that are peculiar with the Sarawak indigenous populations.

## Figures and Tables

**Table 1:** Sociodemographics of the respondents (*N* = 155).

	*n* (%)	Mean (SD)
Age (year)		45.10 (10.233)
Total household income (RM)		817.04 (1054.10)
Race		
Malay	38 (24.5)	
Bidayuh	69 (44.5)	
Iban	48 (31.0)	
Religion		
Islam	41 (26.5)	
Christian	110 (71.0)	
Others	4 (2.6)	
Occupation		
Government	10 (6.5)	
Private	25 (16.1)	
Self-employed	24 (15.5)	
Housewife	90 (58.1)	
Unemployed	6 (3.9)	

**Table 2:** Health profile of the respondents (*N* = 155).

	All		Malay (*n* = 36)	Bidayuh (*n* = 69)	Iban (*n* = 48)	*p* value	Male (*n* = 34)	Female (*n* = 121)	*p* value
	Mean (SD)	*n* (%)	Mean (SD)/*n* (%)				Mean (SD)/*n* (%)		
Atherogenic Index of Plasma (AIP)	0.20 (0.503)		0.14 (0.565)	0.09 (0.395)	0.41 (0.535)		0.51 (0.566)	0.12 (0.450)	
Low (below 0.1)		69 (44.5)	19 (50.0)	38 (55.1)	12 (25.0)	0.004^*∗∗*^	9 (26.5)	60 (49.6)	0.019^*∗∗*^
Intermediate and high (0.1 and higher)		86 (55.5)	19 (50.0)	31 (44.9)	36 (75.0)		25 (73.5)	61 (50.4)	
Physical activity (MET)	9979.13 (9611.189)		8574.89 (7314.260)	9043.53 (8783.824)	12437.69 (11826.99)		13606.34 (11280.637)	8960.69 (8875.796)	
Low and moderate		45 (29.0)	9 (23.7)	19 (27.5)	17 (35.4)	0.460	8 (23.5)	37 (30.6)	0.523
High		110 (71.0)	29 (76.3)	50 (72.5)	31 (64.6)		26 (76.5)	84 (69.4)	
BMI (kg/m^2^)	29.80 (4.006)		30.23 (4.281)	29.68 (3.722)	29.62 (4.231)		28.46 (2.719)	30.17 (4.232)	
Overweight		5 (3.2)	2 (5.3)	2 (2.8)	1 (2.1)	0.606	1 (2.9)	4 (3.3)	0.859
Obese		150 (96.8)	36 (94.7)	67 (97.2)	47 (97.9)		33 (97.1)	117 (96.7)	
% of body fat	37.53 (9.963)		38.76 (8.091)	37.54 (11.237)	36.56 (9.425)		27.97 (5.636)	40.22 (9.242)	
Overfat		148 (95.5)	38 (100.0)	64 (92.8)	46 (95.8)	0.223	32 (94.1)	116 (95.9)	0.648
Blood pressure									
Systolic	131.07 (16.319)		134.13 (16.917)	129.38 (17.220)	131.08 (14.369)		134.21 (15.019)	130.19 (16.618)	
Diastolic	77.17 (10.175)		79.13 (9.253)	76.88 (10.802)	76.04 (9.923)		77.79 (11.860)	77.00 (9.698)	
Hypertension		27 (17.4)	12 (31.6)	7 (10.1)	8 (16.7)	0.020^*∗∗*^	7 (20.6)	20 (16.5)	0.612
Cholesterol (mmol/dL)	5.58 (0.998)		5.88 (1.074)	5.55 (0.878)	5.37 (1.057)		5.50 (0.930)	5.60 (1.018)	
Hypercholesterolemia		102 (65.8)	29 (76.3)	46 (66.7)	27 (56.3)	0.147	22 (64.7)	80 (66.1)	0.516
LDL (mmol/dL)	1.32 (0.316)		3.59 (1.138)	2.67 (1.131)	2.95 (1.294)		2.98 (1.255)	2.98 (1.232)	
HDL (mmol/dL	2.98 (1.233)		1.41 (0.264)	1.36 (0.369)	1.21 (0.235)		1.23 (0.309)	1.35 (0.313)	
Glucose (mmol/dL)	5.96 (2.458)		6.27 (1.844)	5.63 (1.844)	6.17 (3.452)		6.07 (2.020)	5.93 (2.574)	
Hyperglycemia		151 (99.4)	38 (100.0)	68 (98.6)	48 (100.0)	0.534	34 (100.0)	120 (99.2)	0.781
Triglyceride	1.78 (1.017)		1.79 (0.979)	1.58 (0.780)	2.07 (1.268)		2.29 (1.357)	1.64 (0.854)	

^*∗∗*^ *p* < 0.01.

**Table 3:** Logistic regression^a^ predicting cardiovascular risk factors among respondents (*N* = 155).

	AIP OR (95% CI)	Physical activity OR (95% CI)	Body fat	Hypertension OR (95% CI)	Hypercholesterolemia OR (95% CI)	Hyperglycemia OR (95% CI)
Race						
Malay (Ref.)						
Bidayuh	0.30 (0.12, 0.78)^*∗*^	1.98 (0.74, 5.30)	4.9 (1.22, 7.89)	1.96 (0.68, 5.63)	2.10 (0.80, 5.54)	2.04 (0.77, 4.98)
Iban	0.29 (0.12, 0.69)^*∗∗*^	1.75 (0.74, 4.10)	0.33 (0.05, 2.28)	0.48 (0.15, 1.53)	1.33 (0.59, 3.02)	3.86 (1.06, 6.66)
Gender						
Male (Ref.)						
Female	2.05 (0.83, 5.03)	1.68 (0.66, 4.29)	0.36 (0.05, 2.45)	0.92 (0.33, 2.58)	0.905 (0.38, 2.16)	4.14 (0.85, 4.75)

^a^Adjusted for age, ^*∗*^ *p* < 0.05, and ^*∗∗*^ *p* < 0.01.

## References

[1] World Health Organization (WHO) Cardiovascular diseases (CVDs) Fact sheet 2015. http://www.who.int/mediacentre/factsheets/fs317/en/.

[2] Després J.-P. (2012). Body fat distribution and risk of cardiovascular disease: an update. *Circulation*.

[3] Després J.-P., Moorjani S., Lupien P. J., Tremblay A., Nadeau A., Bouchard C. (1990). Regional distribution of body fat, plasma lipoproteins, and cardiovascular disease. *Arteriosclerosis, Thrombosis, and Vascular Biology*.

[4] Ministry of Health Malaysia (1996). *The Second National Health and Morbidity Survey (NHMS II)*.

[5] Ministry of Health Malaysia (2006). *The Third National Health and Morbidity Survey (NHMS III)*.

[6] Chang C. T., Lee P. Y., Cheah W. L. (2012). The prevalence of cardiovascular risk factors in the young and middle-aged rural population in Sarawak, Malaysia. *Malaysian Journal of Medical Sciences*.

[7] Sharp T. A., Grunwald G. K., Giltinan K. E. K., King D. L., Jatkauskas C. J., Hill J. O. (2003). Association of anthropometric measures with risk of diabetes and cardiovascular disease in Hispanic and Caucasian adolescents. *Preventive Medicine*.

[8] Gasperino J. (1996). Ethnic differences in body composition and their relation to health and disease in women. *Ethnicity and Health*.

[9] Goran M. I. (2008). Ethnic-specific pathways to obesity-related disease: the Hispanic vs. African-American Paradox. *Obesity*.

[10] Rampal S., Mahadeva S., Guallar E. (2012). Ethnic differences in the prevalence of metabolic syndrome: results from a multi-ethnic population-based survey in Malaysia. *PLoS ONE*.

[11] Sarawak Tourism Federation, 2016, http://www.stf.org.my/sarawak/index.php?do= people

[12] Chu A. H. Y., Moy F. M. (2015). Reliability and validity of the malay international physical activity questionnaire (IPAQ-M)among a malay population in Malaysia. *Asia-Pacific Journal of Public Health*.

[13] Frohlich J., Dobiášová M. (2003). Fractional esterification rate of cholesterol and ratio of triglycerides to HDL-cholesterol are powerful predictors of positive findings on coronary angiography. *Clinical Chemistry*.

[14] Holmes D. T., Frohlich J., Buhr K. A. (2008). The concept of precision extended to the atherogenic index of plasma. *Clinical Biochemistry*.

[15] National Cholesterol Education Programme Adult Treatment Panel III (2001). *Report of the Expert Panel on the Detection, Evaluation and Treatment of High Cholesterol in Adults*.

[16] Ministry of Health Malaysia (2009). *Clinical Practice Guidelines on Management of Type 2 Diabetes Mellitus*.

[17] Zalesin K. C., Franklin B. A., Miller W. M., Peterson E. D., McCullough P. A. (2011). Impact of obesity on cardiovascular disease. *Medical Clinics of North America*.

[18] Niroumand S., Khajedaluee M., Khadem-Rezaiyan M. (2015). Atherogenic Index of Plasma (AIP): a marker of cardiovascular disease. *Medical Journal of the Islamic Republic of Iran*.

[19] Dobiášová M., Frohlich J. (2001). The plasma parameter log (TG/HDL-C) as an atherogenic index: correlation with lipoprotein particle size and esterification rate inapoB-lipoprotein-depleted plasma (FERHDL). *Clinical Biochemistry*.

[20] Priya K., Desigamini K., Kavitha G., Rita M. A. (2011). Coronary artery diseases. *Journal of Clinical and Diagnostic Research*.

[21] Chang C. T., Chang K. H., Cheah W. L. (2009). Adults' perceptions of being overweight or obese: a focus group study. *Asia Pacific Journal of Clinical Nutrition*.

[22] Hosain G. M. M., Rahman M., Williams K. J., Berenson A. B. (2010). Racial differences in the association between body fat distribution and lipid profiles among reproductive-age women. *Diabetes and Metabolism*.

[23] Roger V. L., Go A. S., Lloyd-Jones D. M. (2011). Heart disease and stroke statistics—2011 update: a report from the American Heart Association. *Circulation*.

[24] Maas A. H. E. M., Appelman Y. E. A. (2010). Gender differences in coronary heart disease. *Netherlands Heart Journal*.

[25] Ford E. S., Capewell S. (2007). Coronary heart disease mortality among young adults in the U.S. From 1980 Through 2002: concealed leveling of mortality rates. *Journal of the American College of Cardiology*.

